# Exercise rather than fluoxetine promotes oligodendrocyte differentiation and myelination in the hippocampus in a male mouse model of depression

**DOI:** 10.1038/s41398-021-01747-3

**Published:** 2021-12-08

**Authors:** Jing Tang, Xin Liang, Xiaoyun Dou, Yingqiang Qi, Chunmao Yang, Yanmin Luo, Fenglei Chao, Lei Zhang, Qian Xiao, Lin Jiang, Chunni Zhou, Yong Tang

**Affiliations:** 1 Department of Histology and Embryology, Faculty of Basic Medical Sciences, Chongqing Medical University, Chongqing, 400016 P. R. China; 2 Laboratory of Stem Cells and Tissue Engineering, Faculty of Basic Medical Sciences, Chongqing Medical University, Chongqing, 400016 P. R. China; 3 Department of Pathologic Physiology, Faculty of Basic Medical Sciences, Chongqing Medical University, Chongqing, 400016 P. R. China; 4 Institute of Life Science, Chongqing Medical University, Chongqing, 400016 P. R. China; 5 Department of Physiology, Faculty of Basic Medical Sciences, Chongqing Medical University, Chongqing, 400016 P. R. China; 6 Department of Radioactive Medicine, Faculty of Basic Medical Sciences, Chongqing Medical University, Chongqing, 400016 P. R. China; 7 Lab Teaching & Management Center, Chongqing Medical University, Chongqing, 400016 P. R. China

**Keywords:** Depression, Molecular neuroscience

## Abstract

Although selective serotonin reuptake inhibitor (SSRI) systems have been meaningfully linked to the clinical phenomena of mood disorders, 15–35% of patients do not respond to multiple SSRI interventions or even experience an exacerbation of their condition. As we previously showed, both running exercise and fluoxetine reversed depression-like behavior. However, whether exercise reverses depression-like behavior more quickly than fluoxetine treatment and whether this rapid effect is achieved via the promotion of oligodendrocyte differentiation and/or myelination in the hippocampus was previously unknown. Sixty male C57BL/6 J mice were used in the present study. We subjected mice with unpredictable chronic stress (UCS) to a 4-week running exercise trial (UCS + RN) or intraperitoneally injected them with fluoxetine (UCS + FLX) to address these uncertainties. At the behavioral level, mice in the UCS + RN group consumed significantly more sugar water in the sucrose preference test (SPT) at the end of the 7th week than those in the UCS group, while those in the UCS + FLX group consumed significantly more sugar water than mice in the UCS group at the end of the 8th week. The unbiased stereological results and immunofluorescence analyses revealed that running exercise, and not fluoxetine treatment, increased the numbers of CC1^+^ and CC1^+^/Olig2^+^/BrdU^+^ oligodendrocytes in the CA1 subfield in depressed mice exposed to UCS. Moreover, running exercise rather than fluoxetine increased the level of myelin basic protein (MBP) and the G-ratio of myelinated nerve fibers in the CA1 subfield in the UCS mouse model. Unlike fluoxetine, exercise promoted hippocampal myelination and oligodendrocyte differentiation and thus has potential as a therapeutic strategy to reduce depression-like behaviors induced by UCS.

## Introduction

Major depressive disorder (MDD) is a recurrent, chronic mental disease that affects millions of individuals worldwide. From 1990 to 2016, MDD rose into the top three disorders on the disease burden list [1]. There is a great need for an improved understanding of the neural mechanisms and pathophysiology of depression and of antidepressant therapeutics. Although selective serotonin reuptake inhibitor (SSRI) systems have been meaningfully linked to the clinical manifestations of mood disorders and the pharmacological treatments employed to treat them over the past 40 years, 15–35% of patients do not respond to multiple interventions or even experience an exacerbation of their condition [2]. However, exercise has been reported to be related to a greater alleviation in depressive patients than any other treatment [3, 4]. In our previous study, we found that running exercise attenuated depression-like behaviors induced by unpredictable chronic stress (UCS) [5, 6]. However, the effectiveness of running exercise and antidepressants for the treatment of depression have not been compared. Therefore, the current study explored the effects of running exercise and fluoxetine on depression-like behavior in a mouse model of depression induced by UCS.

The axonal myelin levels in the corpus callosum splenium are decreased in patients with MDD [7]. The decreased white matter hyperintensities and abnormalities in myelin integrity first occur in prefrontal regions in the early stages of depression as determined by magnetic resonance imaging [8]. Nagy et al. [9] found that the immature oligodendrocyte precursor cells (OPCs) exhibited the most dysregulated genes among all cell types in MDD, with the changes in their gene expression levels accounting for nearly half (47%) of all changes. An animal model of depression induced by chronic stress was shown to exhibit NG2^+^ OPC atrophy and demyelination in the medial prefrontal cortex (mPFC) [10]. Cathomas et al. [11] observed reduced expression of oligodendrocyte-related genes in another animal model of depression subjected to chronic social stress, highlighting oligodendrocytes as a potential target for the treatment of stress-related neuropsychiatric disorders. The findings mentioned above support that myelination and OPC differentiation are involved in the pathogenesis of depressive behaviors. However, the mechanisms of OPCs and myelination in the regulation of depression-like behaviors have not been elucidated. Moreover, whether the generation of new oligodendrocytes or the process of hippocampal remyelination is involved in depression-like behaviors remains unknown.

Although an increasing number of studies have found that impaired oligodendrocyte functions and myelination are involved in the pathogenesis of depressive behaviors, little is known about the mechanisms by which oligodendrocyte production, differentiation, and myelination can be used to treat depression. Recent clinical reports and epidemiological observations have shown that MDD is associated with multiple sclerosis, a neuroinflammatory disorder characterized by the excessive loss of axonal myelin [12]. Clemastine rescued behavioral changes in an animal depression model induced by social isolation and promoted the differentiation and myelination of oligodendrocytes in the mPFC of an animal depression model [13]. Moreover, venlafaxine was shown to successfully attenuate depression-like behaviors in a cuprizone-induced demyelinated mouse model [14]. According to these findings, oligodendrocytes and the subsequent myelination might play an extremely important role in the mechanism of antidepressant therapy. Regarding the correlations between oligodendrocytes and exercise, physical activity was previously shown to promote motor learning, resulting in increased OPC proliferation and oligodendrocyte production in the corpus callosum in the myelin regulatory factor (*Myrf*)^(−/−)^ mice [15]. Regarding the correlations between oligodendrocytes and fluoxetine, Fukushima et al. [16] found that fluoxetine significantly attenuated the LPS-induced decreases in the numbers of bromodeoxyuridine (BrdU)-labeled OPCs in the fornix and corpus callosum. However, Rajkowska et al. [17] did not observe a significant effect of chronic fluoxetine treatment on oligodendrocyte morphometry or the expression of myelin-related mRNAs in rhesus monkeys with MDD. Given these results, the changes occurring in oligodendrocytes and subsequent myelination, which are associated with the antidepressant mechanisms of fluoxetine and exercise, remain unclear. Therefore, we investigated oligodendrocytes at different stages, including OPCs, newborn oligodendrocytes, and mature oligodendrocytes, and detected changes in hippocampal remyelination in UCS model mice subjected to running exercise and fluoxetine treatment. Overall, our findings may lay a foundation for the treatment of depression with exercise and fluoxetine and provide new insights into treatment targets for depression.

## Materials and methods

### Animals and housing

A total of 60 male C57BL/6 J mice (weighing 10–15 g) were housed at a temperature of 22–24 °C under a 12-h/12-h light–dark cycle with free access to water and food. The housing, treatment, and sacrifice procedures for all mice were in accordance with the Guide for the Care and Use of Laboratory Animals of the National Institutes of Health and the Guidelines for the Care and Use of Laboratory Animals of Chongqing Medical University (for details, see the supplementary material).

### UCS paradigm

**Fig. 1:**
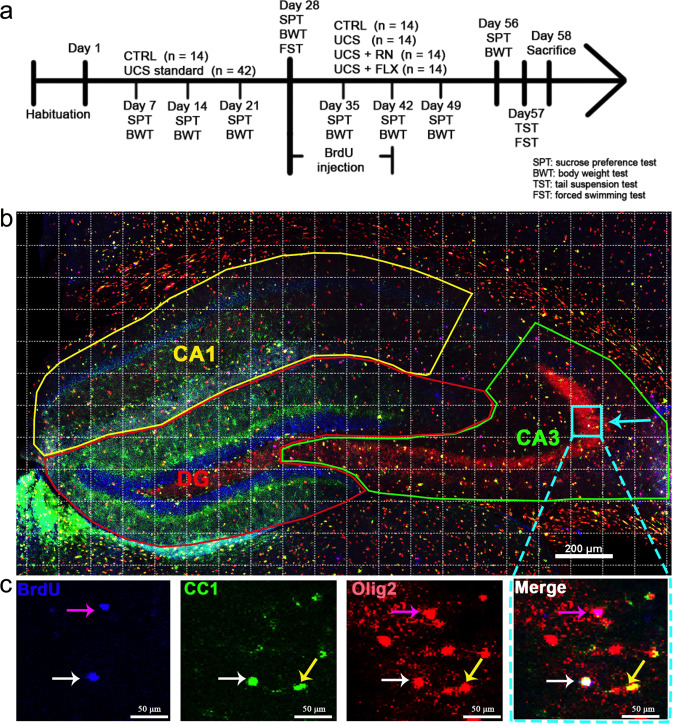
Behavior experiment timeline and illustration of immunofluorescence staining on the whole hippocampus. **a** Behavior experiment timeline. The experiment lasted for 65 days. **b** Immunofluorescence staining for CC1 (green), Olig2 (red), and BrdU (blue) throughout the hippocampus. For quantitative evaluation of co-labeled cells, a rectangular grid was superimposed on the hippocampus, and 12–15 fields (100 × 100 μm^2^) were systematically and randomly sampled from the CA1 (yellow line), CA3 (green line), and DG (red line) subfields of one section. Scale bar = 200 μm. **c** Magnified view of the blue grid indicated by the blue arrow in Fig. 1b. The white arrow indicates a CC1^+^/Olig2^+^/BrdU^+^ co-labeled cell, the pink arrow indicates an Olig2^+^/BrdU^+^ co-labeled cell, and the yellow arrow indicates a CC1^+^/Olig2^+^ co-labeled cell. Scale bar = 50 μm.

The UCS procedure was based on studies by Willner et al. [18], Yohn et al. [19], and Logan et al. [20]. The details are shown in Table 1 and Table 2 (Fig. 1a) (see also the supplementary material).

**Table 1:** Details and abbreviations of the stressors.

Abbreviations	Stressor	Details
Ald	Altered light–dark cycle	30 min alteration of ON/OFF light during the last 3 h of the light phase
Fd	Food deprivation	12 h
Wd	Water deprivation	12 h
cR	Cage rotation	Placement of the cage on a rotating bar for 3 h
cT	Cage tilt	45° for 12 h
Rb	Removed bedding	12 h
Db	Damp bedding	12 h
Ci	Cold isolation	4 °C for 45 min
Res	Restraint	3 h
Str	Stroboscope	12 h in the dark
Fs	Inescapable footshock	0.7 mA for 30 s, stop for 30 s, shock for another 30 s
Ae	Apparatus exposure	Exposure to the footshock apparatus with no footshock for 10 min
Ebs	Empty bottle stress	3 h of water deprivation
Wn	White noise	3 h at 80 decibels
cE	Cage exchange	Mice placed in the cages of other mice for 3 h

**Table 2:** Protocol for UCS.

Day	1	2	3	4	5	6	7	8	9	10	11	12	13	14
	cT	Wn	Res	Ci	Wd	SPT	Wn	cT	Ebs	Ci	Res	cR	SPT	Fd
	cR	Rb	Fd	Ald	Fs	BW	Res	Rb	Str	Wn	Str	Wn	BW	Fs
							cE	Wd	cR	Fd	Ald	Fs		Wn
Day	15	16	17	18	19	20	21	22	23	24	25	26	27	28
	cT	Ebs	Ci	Str	Fs	SPT	Wn	cE	cT	Res	Ebs	cR	SPT	Str
	Db	cR	Wn	Ald	Ci	BW	Fd	cR	Str	Wn	Fs	Str	BW	Fs
	Wd	Res	Fd	cR	Ald		Fs	Res	Ci	Wd	Ald	Ci	FST	Ae
Day	29	30	31	32	33	34	35	36	37	38	39	40	41	42
	FST	Wn	Ebs	Ald	Fs	SPT	Wn	cE	cT	Res	Ebs	Ae	SPT	cE
	TST	cR	Ci	Db	Rb	BW	Fd	cR	Rb	Fs	Str	Wn	BW	Wn
		Wd	Str	cT	Ae		Fs	Res	Ci	Wd	Ald	cR		Res
Day	43	44	45	46	47	48	49	50	51	52	53	54	55	56
	cT	Ae	Wn	Ald	Res	SPT	Fs	Ebs	Res	Str	Ald	Wn	Rb	SPT
	Db	Wd	Ebs	cR	Str	BW	Wd	cT	cR	Ci	Ae	Fd	Res	BW
	Fs	Ci	Str	Ci	Wn		Ci	Rb	cE	Wn	Res	Fs	cT	
Day	57	58												
	FST	S												
	TST													

*SPT* sucrose preference test, *BW* body weight, *FST* forced swimming test, *TST* tail suspension test, *S* sacrifice.

### Exercise protocol

During the first 2 weeks, the running speed was gradually increased from 5 m/min to 10 m/min. For the next 2 weeks, the running speed remained at 10 m/min [21] (for details, see the supplementary material).

### Fluoxetine and BrdU injections

Mice were intraperitoneally injected with fluoxetine (Sigma-Aldrich, USA) at a dosage of 10 mg/kg/d and with BrdU at a dosage of 50 mg/kg/d body weight (BW) [21, 22] (for details, see the supplementary material).

### Behavioral test

The behavioral tests included the body weight measurement (BW), the sucrose preference test (SPT), the forced swimming test (FST), and the tail suspension test (TST). The SPT and FST were performed at the end of the 4th week, and the SPT, FST, and TST were performed at the end of the 8th week (Fig. 1a). The protocols are described in the supplementary material [5, 21].

### Perfusion and tissue preparation

After the behavioral tests, five mice from each group were randomly anesthetized (i.p. injection of 1% pentobarbital sodium) and were perfused with 4% paraformaldehyde through the heart. The brains were removed and divided into the right and left hemispheres through midsagittal sectioning. A randomly sampled hemisphere from each mouse was placed on a cryostat microtome (CM1860, Leica), and every 5th section was maintained in an anatomical series. The sections were stored at −20 °C in 75% ethanol. On average, 15–18 slices containing hippocampal structures were randomly selected from each group for immunohistochemistry and immunofluorescence staining [5] (for details, see the supplementary material).

### Immunohistochemistry and stereological analyses

Two serial sections containing the hippocampus were randomly chosen from each group of mice and immunoreacted with goat anti-PDGFα (1:500; AF1062, R&D Systems) and mouse anti-CC1 (1:500; OP80, EMD Millipore) antibodies for stereological analyses of the total numbers of mature oligodendrocytes and OPCs in the hippocampus. An optical fractionator was used to estimate the total numbers of PDGFα^+^ and CC1^+^ cells in the hippocampus (for details, see the supplementary material and Fig. S1).

### Immunofluorescence analyses

Mice were anesthetized and then perfused, tissues were cryopreserved, embedded, and sectioned as described above. Immunohistochemistry was performed with primary antibodies against PDGFα (1:500; AF1062, R&D Systems), CC1 (1:500; OP80, EMD Millipore), BrdU (1:500; ab6326, Abcam), MBP (1:1000; ab62631, Abcam), and Olig2 (1:500; ab109186, Abcam). The stained sections were visualized by laser scanning confocal microscopy (Nikon Eclipse Ti microscope, NIS-Elements AR Imaging). Quantification of CC1^+^/Olig2^+^/BrdU^+^ and PDGFα^+^/Olig2^+^ cells was performed using the count quantification function of a NIS-Elements AR analysis system. The MBP^+^ areas were quantified using the defined area method of the NIS-Elements AR analysis system (for details, see the supplementary material).

### Transmission electron microscopy (TEM) and G-ratio analysis

Each hemisphere was coronally sectioned into 1-mm-thick serial-parallel blocks. The blocks were randomly sampled at the beginning of the hippocampus. Then, 1-mm^3^ tissue blocks were randomly sampled in the cornu ammonis (CA) 1 region of the hippocampus. Approximately five tissue blocks per mouse were sampled at random. The ultrathin sections were observed in the ‘S’ route mode by TEM (JEM-1400 plus, Hitachi, Japan). Twenty fields in each section were randomly selected and photographed by TEM at a magnification of ×10,000, with 100–125 TEM images being acquired from each group. ImageJ analysis software was used to measure the axons and the outer diameter of each myelinated nerve fiber in the hippocampal CA1 region, and the G-ratio value was calculated using the following formula (for details, see the supplementary material):

$${{{\mathrm{G-ratio}}}} = {{{\mathrm{axon}}}}\;{{{\mathrm{diameter}}}}\;{{{\mathrm{of}}}}\;{{{\mathrm{myelinated}}}}\;{{{\mathrm{nerve}}}}\;{{{\mathrm{fibers}}}}/{{{\mathrm{outer}}}}\;{{{\mathrm{myelin}}}}\;{{{\mathrm{diameter}}}}\;{{{\mathrm{of}}}}\;{{{\mathrm{myelinated}}}}\;{{{\mathrm{nerve}}}}\;{{{\mathrm{fibers}}}}$$

### Statistics

Levene’s test was used to evaluate the similarities of variances among the groups. All data were normally distributed. Then, the BW and SPT data were analyzed using repeated-measures analysis of variance (ANOVA). If Mauchly’s sphericity assumption was met, Mauchly’s test was used; otherwise, the Greenhouse-Geisser test was used. Data from two groups (control [CTRL] and UCS standard) were compared using independent-samples *t* tests. Data from four groups (CTRL, UCS, UCS + running exercise [RN], and UCS + fluoxetine [FLX]) were compared using one-way ANOVA. If the data displayed similar variances among groups, a least significant difference post hoc test was adopted for analysis; otherwise, Tamhane’s post hoc test was performed. The observed coefficient of errors (OCEs) of the CC1^+^ and PDGFα^+^ cells in the hippocampus, along with the OCE divided by the observed coefficient of variation squared (OCV^2^), were calculated as described by Schmitz and Hof [23]. The sample size for each experiment was chosen based on previous experience and aimed to detect at least a *P* < 0.05 in the different tests applied. An OCE value <0.15 and an OCE^2^/OCV^2^ value <0.5 indicated that the sampled animals and the sampled fields of the four groups were sufficient for the stereological analyses. A *P* value <0.05 indicated a significant difference. All the experiments and data analyses were performed blind to treatment conditions.

## Results

### Running exercise rescued the depressive behavior in UCS-exposed mice faster and more efficiently than fluoxetine treatment

**Fig. 2:**
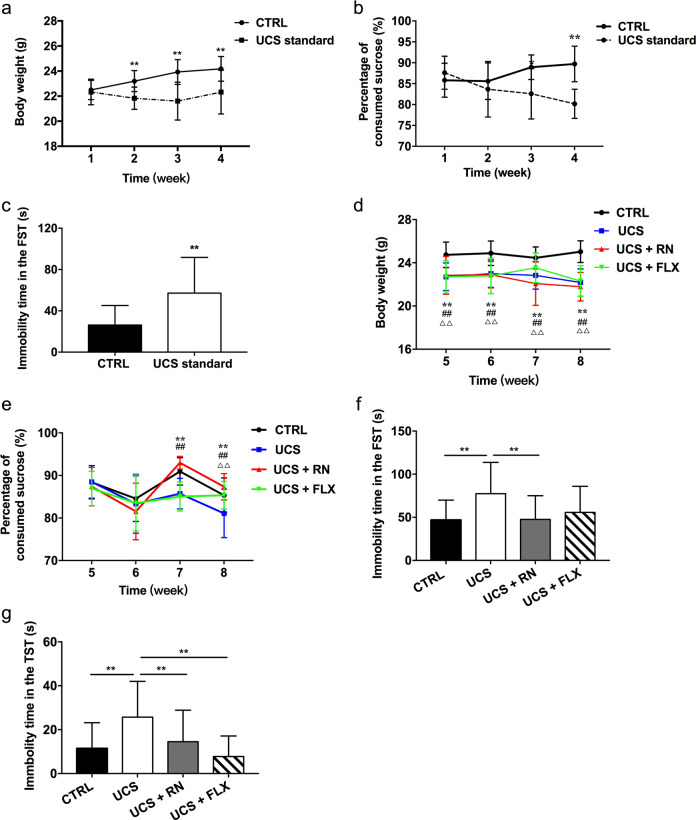
The effects of running exercise and fluoxetine on the depression-like behaviors of UCS-exposed mice. **a** Line graph illustrating the BWs of the mice in the CTRL group (*n* = 14) and the UCS group (*n* = 42) during the first 4 weeks. **b** Line graph illustrating the sucrose preferences of the mice in the CTRL group (*n* = 14) and the UCS group (*n* = 42) during the first 4 weeks. **c** FST immobility time (in seconds) of the mice in the CTRL group (*n* = 14) and the UCS group (n = 42) at the end of the 4th week. **d** Line graph illustrating the BWs of the mice in the CTRL group (*n* = 14), UCS group, UCS + RN group, and UCS + FLX group during the last 4 weeks (*n* = 14 for each group). **indicates *P* < 0.01, UCS group vs. CTRL group. ## indicates *P* < 0.01, UCS + RN group vs. CTRL group. ΔΔ indicates *P* < 0.01, UCS + FLX group vs. CTRL group. **e** Line graph illustrating the sucrose preferences of the mice in the CTRL group, UCS group, UCS + RN group, and UCS + FLX group during the last 4 weeks (*n* = 14 for each group). **indicates *P* < 0.01, UCS group vs. CTRL group. ## indicates *P* < 0.01, UCS + RN group vs. CTRL group. ΔΔ indicates *P* < 0.01, UCS + FLX group vs. CTRL group. **f** FST immobility time (in seconds) of the mice in the CTRL group, UCS group, UCS + RN group, and UCS + FLX group at the end of 8 weeks (*n* = 14 for each group). **g** FST immobility time (in seconds) of the mice in the CTRL group, UCS group, UCS + RN group, and UCS + FLX group at the end of the 8th week (*n* = 14 for each group). All data are shown as the means ± SDs, * *P* < 0.05, and ** *P* < 0.01.

Mice were subjected to UCS for 4 weeks to induce depression-like behavior (Fig. 2a–c). At the end of 4th week, the BW of the UCS standard group was significantly lower than that of the CTRL group (*P* = 0.009) (Fig. 2a). The sucrose preference percentage in the UCS standard group was significantly lower than that in the CTRL group at the end of the 4th week (*P* < 0.001) (Fig. 2b). The immobility time of the UCS standard group in the FST was longer than that of the CTRL group (*P* = 0.002) (Fig. 2c). These results indicated the successful establishment of the UCS-induced depression model in the mice.

At the end of the 8th week, the BWs of the UCS, UCS + RN, and UCS + FLX groups were still significantly lower than that of the CTRL group (*P* = 0.012 for UCS vs. CTRL, *P* = 0.026 for UCS vs. UCS + FLX) (Fig. 2d). At the end of the 7th week, the sucrose preference percentage in the UCS + RN group was significantly increased compared with that in the UCS group (*P* < 0.001). However, the sucrose preference percentage did not significantly differ between the UCS + FLX and UCS groups (*P* = 0.557) (Fig. 2e). At the end of the 8th week, the sucrose preference percentages of the mice in the UCS + RN and UCS + FLX groups were significantly greater than that of the mice in the UCS group (*P* < 0.001 for UCS vs. UCS + RN, *P* = 0.009 for UCS vs. UCS + FLX) (Fig. 2e). Additionally, the immobility times in the TST of the mice in the UCS + RN and UCS + FLX groups were shorter than those of the mice in the UCS group (*P* = 0.005 for UCS vs. UCS + RN, *P* < 0.001 for UCS vs. UCS + FLX). In contrast, the immobility time in the FST of the mice in the UCS + RN group was significantly shorter than that of the mice in the UCS group, but the times were not significantly different between the UCS + FLX group and the UCS group (*P* = 0.01 for UCS vs. UCS + RN, *P* = 0.057 for UCS vs. UCS + FLX) (Fig. 2f, g). These results indicated that the exercise treatment was faster and more efficient than fluoxetine treatment at reversing depressive behavior in UCS mice.

### The number of hippocampal OPCs was increased in UCS-exposed mice, and the increase was reversed by both running exercise and fluoxetine treatment

**Fig. 3:**
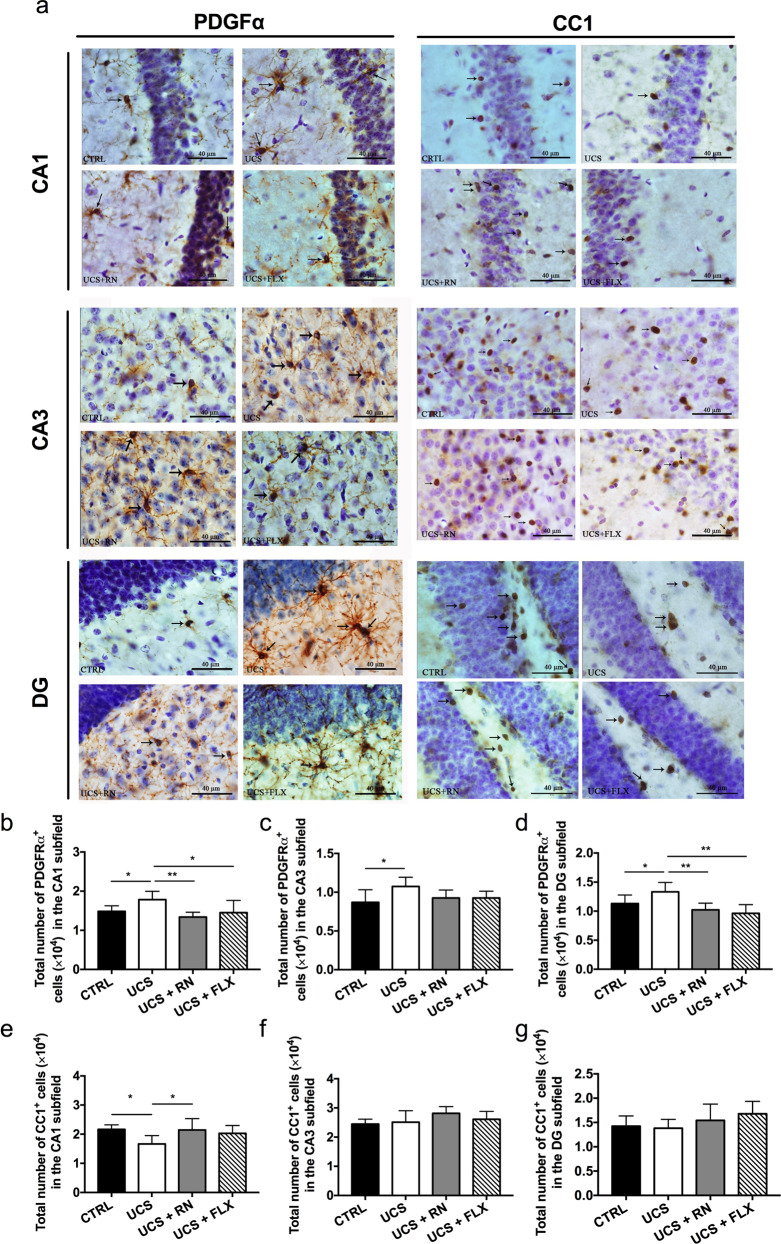
The effects of running exercise and fluoxetine on the mature oligodendrocytes and OPCs of the hippocampus. **a** Immunohistochemical staining with an anti-PDGFα antibody and anti-CC1 antibody in the CA1, CA3, and DG hippocampal subfields of mice in the CTRL group, UCS group, UCS + RN group, and UCS + FLX group. The black arrows indicate the PDGFα^+^ cells or the CC1^+^ cells. Scale bar = 40 μm. **b**–**d** The numbers of PDGFα^+^ OPCs in the CA1, CA3, and DG hippocampal subfields of mice in the CTRL group, UCS group, UCS + RN group, and UCS + FLX group (*n* = 5 for each group). **e**–**g** Stereological analyses of the CC1^+^ cells in the CA1, CA3, and DG hippocampal subfields of mice in the CTRL group, UCS group, UCS + RN group, and UCS + FLX group (*n* = 5 for each group). All data are shown as the means ± SDs, * *P* < 0.05 and **P < 0.01.

To assess the differences in OPC numbers among the CTRL, UCS, UCS + RN, and UCS + FLX groups, we analyzed the numbers of PDGFα^+^ oligodendrocytes in the three hippocampal subfields. The results of immunohistochemical staining with an anti-PDGFα antibody in the CA1, CA3, and dentate gyrus (DG) subfields of hippocampi from the CTRL, UCS, UCS + RN, and UCS + FLX groups are presented in Fig. 3a. The analyses of the sampling design used to estimate the numbers of PDGFα^+^ cells in the CA1, CA3, and DG regions of the hippocampus via unbiased stereological analyses are illustrated in Table S1. The numbers of OPCs were significantly increased in the CA1 (*P* = 0.037) (Fig. 3b), CA3 (*P* = 0.017) (Fig. 3c) and DG (*P* = 0.039) (Fig. 3d) hippocampal subfields of mice in the UCS group compared with CTRL group mice. Moreover, the numbers of PDGFα^+^ OPCs in the CA1 (*P* = 0.004 for UCS vs. UCS + RN, *P* = 0.024 for UCS vs. UCS + FLX) (Fig. 3b) and DG hippocampal subfields (*P* = 0.003 for UCS vs. UCS + RN, *P* = 0.001 for UCS vs. UCS + FLX) of mice in the UCS + RN and UCS + FLX groups were significantly lower than those in these hippocampal regions of UCS group mice (Fig. 3d). The numbers of PDGFα^+^ cells in the CA3 subfield were not significantly different among the UCS, UCS + RN and UCS + FLX groups (*P* = 0.088) (Fig. 3c).

### The number of mature oligodendrocytes was decreased in only the hippocampal CA1 region in UCS-exposed mice, and only running exercise (not fluoxetine) reversed the decrease

To assess differences in the numbers of mature oligodendrocytes among the CTRL, UCS, UCS + RN, and UCS + FLX groups, we analyzed the numbers of mature oligodendrocytes in the three hippocampal subfields. The results of immunohistochemical staining with an anti-CC1 antibody in the CA1, CA3, and DG hippocampal subfields of mice in the CTRL, UCS, UCS + RN, and UCS + FLX groups are presented in Fig. 3a. The mean total number of cells positive for CC1, a marker for mature oligodendrocytes, and the analyses of the sampling design used to estimate the numbers of CC1^+^ cells in the CA1, CA3, and DG hippocampal regions via unbiased stereological analyses are illustrated in Table S2. In contrast to the PDGFα^+^ cell numbers, the number of CC1^+^ cells was significantly decreased in the CA1 subfield in the UCS-exposed mice (*P* = 0.014) (Fig. 3e); however, the CC1^+^ cell numbers in the CA3 (*P* = 0.72) (Fig. 3f) and DG subfields (*P* = 0.80) (Fig. 3g) were not significantly different between UCS-exposed mice and CTRL mice. Moreover, the number of CC1^+^ cells in the CA1 subfield was significantly greater in the UCS + RN group than in the UCS group, whereas the numbers in the CA1 subfield were not significantly different between the UCS + FLX group and the UCS group (*P* = 0.018 for UCS vs. UCS + RN, *P* = 0.062 for UCS vs. UCS + FLX) (Fig. 3e). No significant differences in the numbers of CC1^+^ cells in the CA3 (*P* = 0.215) (Fig. 3f) and DG subfields (*P* = 0.483) were detected among the UCS, UCS + RN and UCS + FLX groups (Fig. 3g).

### Running exercise rather than fluoxetine enhanced the oligodendrocyte differentiation in the CA1 subfield in UCS-exposed mice

**Fig. 4:**
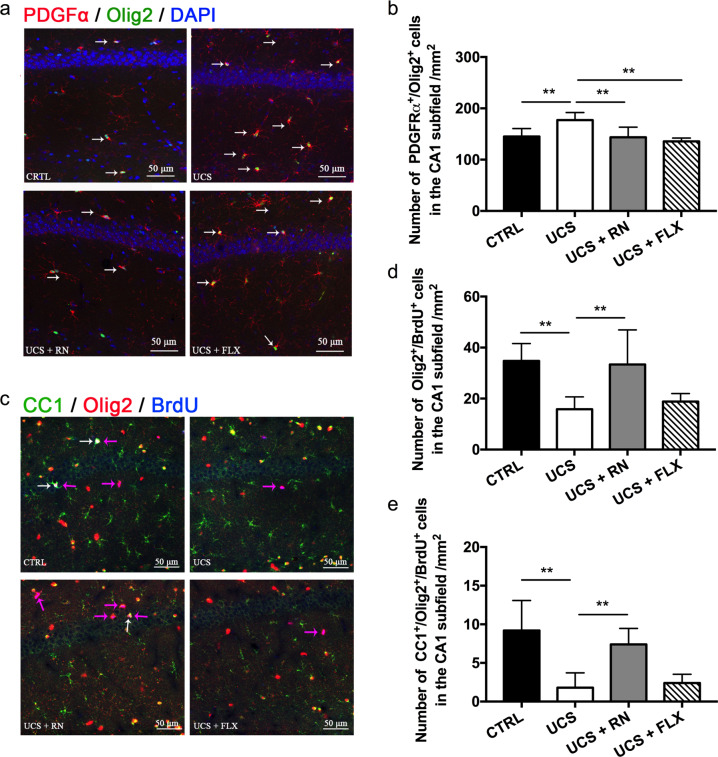
The effects of running exercise and fluoxetine on the OPC proliferation and differentiation in the CA1 subfield of the hippocampus. **a** Colocalization of PDGFα^+^ and Olig2^+^ cells in the CA1 subfield of the hippocampus. PDGFα^+^: red, Olig2^+^: green, DAPI: blue. The white arrows indicate PDGFα^+^/Olig2^+^/DAPI co-labeled cells. Scale bar = 50 μm. **b** Immunofluorescence analyses of the PDGFα^+^/Olig2^+^ cells in the CA1 subfield of mice in the CTRL group, UCS group, UCS + RN group, and UCS + FLX group (*n* = 5 for each group). **c** Colocalization of CC1^+^, Olig2^+^, and BrdU^+^ cells in the CA1 subfield of the hippocampus. CC1^+^: red, Olig2^+^: green, BrdU^+^: blue. The white arrows indicate the CC1^+^/Olig2^+^/BrdU^+^ co-labeled cells, and the pink arrows indicate the Olig2^+^/BrdU^+^ co-labeled cells. Scale bar = 50 μm. **d**, **e** Immunofluorescence analyses of the BrdU^+^/Olig2^+^ and CC1^+^/BrdU^+^/Olig2^+^ cells in the CA1 subfield of mice in the CTRL group, UCS group, UCS + RN group, and UCS + FLX group (*n* = 5 for each group). All data are shown as the means ± SDs. ** *P* < 0.01.

Whether the increase in OPCs in the UCS group was due to increased OPC proliferation, increased OPC differentiation or a combination of both remained unclear. Olig2, a key fate-determining transcription factor of the oligodendrocyte lineage, has been implicated as a driver of neural stem cell proliferation [24]. The number of PDGFα^+^/Olig2^+^ cells in the CA1 subfield was significantly higher in the UCS group than in the CTRL group but significantly lower in the UCS + RN and UCS + FLX groups compared to the UCS group (*P* = 0.004 for CTRL vs. UCS, *P* = 0.003 for UCS vs. UCS + RN, *P* < 0.001 for UCS vs. UCS + FLX) (Fig. 4a, b). BrdU was injected at the end of 4th week to label dividing adult OPCs (Fig. 1b, c); labeling with both BrdU and Olig2 indicated differentiating oligodendrocytes. The number of BrdU^+^/Olig2^+^ cells in the CA1 subfield was significantly lower in the UCS group than in the CTRL group but significantly higher in the UCS + RN group than in the UCS group. The numbers of oligodendrocytes in the CA1 subfield were not significantly different between the UCS group and the UCS + FLX group (*P* = 0.002 for CTRL vs. UCS, *P* = 0.003 for UCS vs. UCS + RN, *P* = 0.414 for UCS vs. UCS + FLX) (Fig. 4c, d). The number of CC1^+^/Olig2^+^/BrdU^+^ cells in the CA1 subfield was substantially lower in the UCS group than in the CTRL group, indicating that chronic stress prevented adult OPCs in the hippocampus from differentiating into mature oligodendrocytes. The number of CC1^+^/Olig2^+^/BrdU^+^ cells in the CA1 subfield was significantly higher in the UCS + RN group than in the UCS group but did not differ between the UCS and UCS FLX groups (*P* < 0.001 for CTRL vs. UCS, *P* = 0.003 for UCS vs. UCS + RN, *P* = 0.707 for UCS vs. UCS + FLX) (Fig. 4c and e).

### Both running exercise and fluoxetine decreased the oligodendrocyte proliferation in the hilus of the DG subfield in UCS-exposed mice

**Fig. 5:**
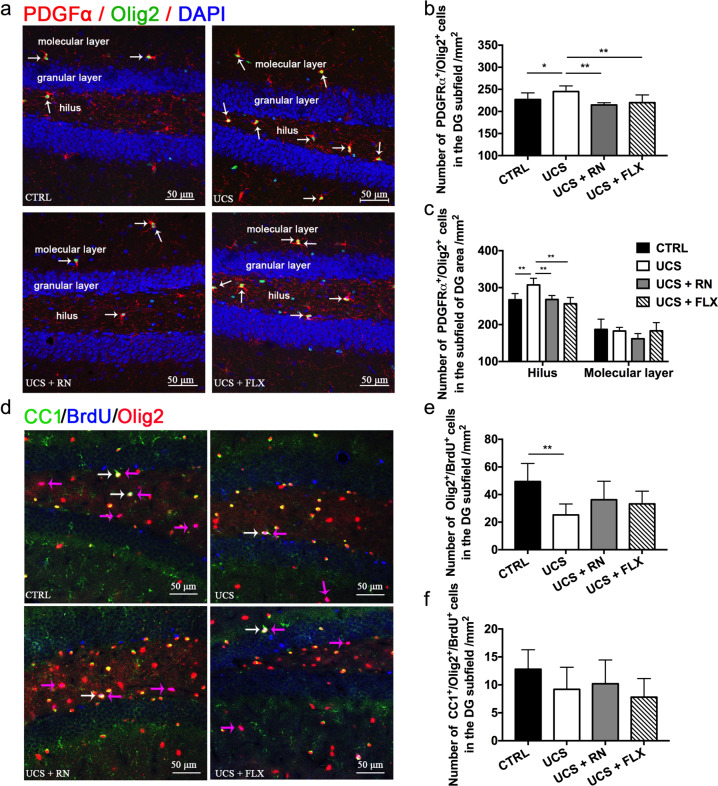
The effects of running exercise and fluoxetine on the OPC proliferation and differentiation in the DG subfield of the hippocampus. **a** Colocalization of PDGFα^+^ and Olig2^+^ cells in the DG hippocampal subfield. PDGFα^+^: red, Olig2^+^: green, DAPI: blue. The white arrows indicate the PDGFα^+^/Olig2^+^/DAPI co-labeled cells. Scale bar = 50 μm. **b** The number of PDGFα^+^/Olig2^+^ OPCs in the DG subfield of mice in the CTRL group, UCS group, UCS + RN group, and UCS + FLX group (*n* = 5 for each group). **c** Immunofluorescence analyses of the PDGFα^+^/Olig2^+^ cells in the hilus and molecular layer of mice in the CTRL group, UCS group, UCS + RN group, and UCS + FLX group (*n* = 5 for each group). **d** Colocalization of the CC1^+^, Olig2^+^, and BrdU^+^ cells in the DG subfield of the hippocampus. CC1^+^: red, Olig2^+^: green, BrdU^+^: blue. The white arrows indicate the CC1^+^/Olig2^+^/BrdU^+^ co-labeled cells, and the pink arrows indicate the Olig2^+^/BrdU^+^ co-labeled cells. Scale bar = 50 μm. **e**–**f** Immunofluorescence analyses of the BrdU^+^/Olig2^+^ and CC1^+^/BrdU^+^/Olig2^+^ cells in the DG subfield of mice in the CTRL group, UCS group, UCS + RN group, and UCS + FLX group (*n* = 5 for each group). All data are shown as the means ± SDs. * *P* < 0.05 and **P < 0.01.

Immunohistochemical analyses revealed more PDGFα^+^/Olig2^+^ cells in the DG subfield in the UCS group than in the CTRL group. Significantly fewer PDGFα^+^/Olig2^+^ cells in the DG subfield were observed in the UCS + RN and UCS + FLX groups than in the UCS group (*P* = 0.05 for CTRL vs. UCS, *P* = 0.003 for UCS vs. UCS + RN, *P* = 0.009 for UCS vs. UCS + FLX) (Fig. 5a, b). The DG subfield contains the hilus and molecular layer, and we herein calculated the PDGFα^+^/Olig2^+^ cell densities in these DG structures. More PDGFα^+^/Olig2^+^ cells were observed in the hilus of the DG subfield in the UCS group than in the CTRL group. Significantly fewer PDGFα^+^/Olig2^+^ cells were observed in the hilus of the DG subfield in the UCS + RN and UCS + FLX groups than in the UCS group (*P* = 0.001 for CTRL vs. UCS, *P* = 0.001 for UCS vs. UCS + RN, *P* = 0.07 for UCS vs. UCS + FLX) (Fig. 5a and c). In contrast, the PDGFα^+^/Olig2^+^ cell numbers in the molecular layer of the DG subfield did not differ among the CTRL, UCS, UCS + RN and UCS + FLX groups (*P* = 0.741 for CTRL vs. UCS, *P* = 0.112 for UCS vs. UCS + RN, *P* = 0.962 for UCS vs. UCS + FLX) (Fig. 5a and c). However, the number of BrdU^+^/Olig2^+^ cells in the DG subfield was significantly lower in the UCS group than in the CTRL group, whereas no significant differences in these numbers were observed among the UCS, UCS + RN, and UCS + FLX groups (*P* *= 0.003 for CTRL vs. UCS, P* *=* *0.139 for UCS vs. UCS* *+* *RN, P* *=* *0.274 for UCS vs. UCS* *+* *FLX*) (Fig. 5d, e). Moreover, no significant differences in the CC1^+^/Olig2^+^/BrdU^+^ cell numbers in the DG subfield were observed among the CTRL, UCS, UCS + RN, and UCS + FLX groups (*P* = 0.239) (Fig. 5d and f).

### Running exercise rather than fluoxetine enhanced myelination in only the CA1 subfield in UCS-exposed mice

**Fig. 6:**
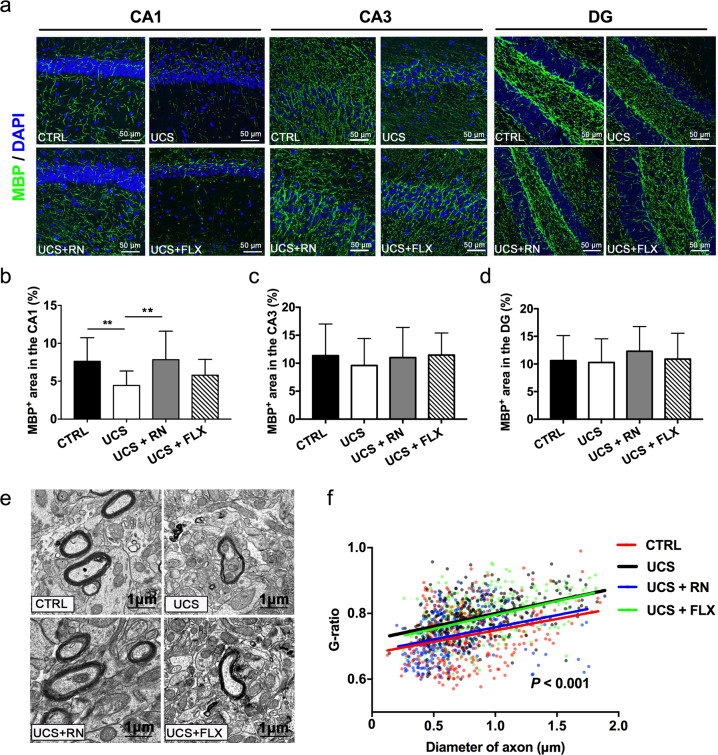
The effects of running exercise and fluoxetine on the myelination in the hippocampus. **a** Immunohistochemical staining for MBP^+^ (green) myelinated fibers in the CA1, CA3, and DG subfields of the hippocampus. DAPI (blue) was used as a nuclear counterstain. Scale bar = 50 μm. **b**–**d** Immunofluorescence analyses of MBP^+^ (green) myelinated fibers in the CA1, CA3, and DG hippocampal subfields of mice in the CTRL group, UCS group, UCS + RN group, and UCS + FLX group (*n* = 5 for each group). **e** Electron micrographs of axons in the CA1 hippocampal subfield of mice in the CTRL group, UCS group, UCS + RN group, and UCS + FLX group. Scale bar = 1 μm. **f** Quantification of the myelin sheath thickness (G-ratio) in the CA1 subfield of mice in the CTRL group (*n* = 303 axons from four mice), UCS group (*n* = 316 axons from four mice), UCS + RN group (*n* = 332 axons from four mice), and UCS + FLX group (*n* = 310 axons from four mice). All data are shown as the means ± SDs. * *P* < 0.05 and ** *P* < 0.01.

Immunofluorescence staining was performed in the CA1, CA3, and DG hippocampal subfields of mice from the CTRL, UCS, UCS + RN, and UCS + FLX groups (Fig. 6a). The percentage of MBP^+^ myelinated fibers in the CA1 subfield was significantly lower in mice from the UCS group than in mice from the CTRL group, while it was significantly higher in mice of the UCS + RN group than in those of the UCS group; however, the numbers did not differ between the UCS group and the UCS + FLX group (*P* < 0.001 for CTRL vs. UCS, *P* < 0.001 for UCS vs. UCS + RN, *P* = 0.07 for UCS vs. UCS + FLX) (Fig. 6b). Moreover, immunohistochemical analysis of the percentages of MBP^+^ myelinated fibers in the DG (*P* = 0.054) and CA3 (*P* = 0.491) subfields revealed no differences among the CTRL, UCS, UCS + RN, and UCS + FLX groups (Fig. 6c, d). Ultrastructural analyses revealed a restoration of myelin thickness in the CA1 subfield in UCS-exposed mice treated with running exercise and fluoxetine to levels comparable to those in the UCS group (Fig. 6e). The G-ratio results revealed that the myelin sheath in the UCS group was thicker than that in the CTRL group. This change was reversed when the UCS-exposed mice were subjected to running exercise. The G-ratios of the UCS and UCS + FLX groups were similar (*P* < 0.001 for CTRL vs. UCS, *P* < 0.001 for UCS vs. UCS + RN, *P* = 0.834 for UCS vs. UCS + FLX) (Fig. 6f).

## Discussion

In summary, the results of this study showed that (1) 3 weeks of the running exercise was beneficial for reversing the sucrose preferences of UCS-exposed mice in the SPT, whereas 3 weeks of fluoxetine treatment failed to reverse the SPT sucrose preferences of UCS-exposed mice. (2) The number of mature oligodendrocytes was decreased in only the CA1 region of the hippocampus in UCS-exposed mice, and running exercise rather than fluoxetine reversed the decrease in the number of mature oligodendrocytes. The number of OPCs has increased in the CA1 and DG hippocampal subfields in UCS-exposed mice, and both running exercise and fluoxetine reversed the increase in the OPC numbers. (3) Both running exercise and fluoxetine enhanced the oligodendrocyte proliferation in the DG subfield hilus in UCS-exposed mice. However, only running exercise enhanced the oligodendrocyte differentiation, maturation, and myelination in the CA1 subfield of UCS-exposed mice.

An unexpected observation was that 3 weeks of the running exercise was beneficial for reversing the sucrose preferences of UCS-exposed mice in the SPT, whereas 3 weeks of fluoxetine treatment failed to reverse the SPT sucrose preferences of UCS-exposed mice. In a clinical study, 12 weeks of fluoxetine treatment did not reduce depressive symptoms in young people with moderate-to-severe MDD [25]. In a preclinical study, Wen et al. [26] found that UCS evoked anhedonia in mice and that both aerobic exercise and fluoxetine reduced the severity of anhedonia at 28 days (the 4th week). However, in the study by Wen et al., the SPT was conducted only on days 10 and 28, and the authors did not observe a difference in onset time between the running exercise group and the fluoxetine group. Unlike Wen and colleagues, we subjected mice in the CTRL, UCS, UCS + RN, and UCS + FLX groups to the SPT at the end of every week. At the end of the 7th week, running exercise rather than fluoxetine was found to be beneficial for reversing depression-like behavior in UCS-exposed mice. In addition, at the end of the 8th week, both running exercise and fluoxetine reversed the behaviors of UCS-exposed mice in the SPT and TST, but only running exercise reversed the change in the FST immobility time for the UCS-exposed mice. The FST and TST are widely performed in animal models of depression to screen for potential antidepressants [27]. Advantageously, the FST is highly accurate and sensitive for evaluating antidepressant effects [28]. Genetic ablation of the 11β-hydroxysteroid dehydrogenase (11β-HSD1) gene was found to result in an antidepressant-like phenotype in the FST but not in the related TST [29]. Although the TST and FST share a common theoretical basis, they have many differences and might therefore complement each other when used to evaluate antidepressant effects under some conditions [30]. Therefore, we conclude that running exercise rescues depressive behaviors in UCS-exposed mice faster and more efficiently than fluoxetine.

Which kinds of cells participate in the underlying mechanism of this superior effect? Many studies suggest that neurogenesis, microglia, and astrocytes play important roles in the pathogenesis of depression [31–33]. In particular, neurogenesis was found to be involved in the antidepressant activity of not only fluoxetine but also running exercise [33–37]. However, these studies investigated the effects of fluoxetine and running exercise on depressed mice and found that both treatments showed similar effects in terms of increasing neurogenesis and the dendritic spine density within 4 weeks [34, 37]. Moreover, fluoxetine begins to slightly increase the proliferation of BrdU^+^ cells in the hippocampi of normal rats after 5 days [38], and the increase becomes significant after 7 days [39]. Voluntary physical exercise is a very effective method for inducing adult neurogenesis in the DG [40, 41]. Increased proliferation of neuronal (DCX^+^/NeuN^+^) cells was also observed in the hippocampi of normal rats after 7 days of treadmill running [42, 43]. Together, these data suggest that fluoxetine treatment and running exercise promote neurogenesis in the hippocampus. However, it is highly likely that the promotion of neurogenesis is not the main mechanism by which running exercise reverses depression-like behavior faster and more efficiently than fluoxetine treatment in UCS mice. Mandyam and colleagues found that adult male rats that voluntarily exercised did not exhibit altered neurogenesis (BrdU^+^/NeuN^+^ cells) but displayed enhanced mPFC gliogenesis (BrdU^+^/GFAP^+^ and BrdU^+^/NG2^+^ cells) [44]. Similarly, McKenzie et al. [15] found that running exercise promoted motor learning, thereby increasing oligodendrocyte production and OPC proliferation in the white matter of *Myrf*-knockout mice. Thus, we speculated that running exercise reversed depression-like behavior in UCS-exposed mice faster and more efficiently than fluoxetine by affecting the changes of oligodendroglial lineage cells.

How does exercise promote these changes in oligodendrocyte biology and myelination, and how does this subsequently impact MDD etiology? In both clinical and preclinical studies, the researchers reported that the genes that are important for differentiation, maturation of oligodendrocytes, and myelin structure (miR-92a-3p, NRG1, FGF2, MAG, MAL, PMP22, PLLP, PLP1) were significantly downregulated in MDD and suicide. Therefore, they thought that oligodendroglial lineage cells were involved in MDD and suicide [12, 45]. In our study, oligodendrocytes at the proliferation and differentiation stages were assayed by double/triple immunofluorescence staining and laser confocal microscopy. Olig2^+^/PDGFα^+^ staining indicated oligodendroglial cells in the proliferation phase, BrdU^+^/Olig2^+^ staining indicated oligodendroglial cells in the differentiation phase, and BrdU^+^/Olig2^+^/CC1^+^ staining indicated oligodendroglial cells in the newborn mature phase. In the current study, the proliferation of OPCs was increased while the differentiation of oligodendrocytes and newborn mature oligodendrocytes was decreased in the hippocampi of depression model mice (Fig. 4d, e). However, running exercise reversed these changes in the maturation and differentiation of oligodendrocytes. Therefore, we speculate that the differentiation and maturation of oligodendrocytes play an important role in the pathogenesis of depression. Myelin is formed by mature oligodendrocytes in the central nervous system [46]. When demyelination occurs in one brain area, OPCs terminally differentiate into premyelinating oligodendrocytes, which further mature and myelinate near receptive axons [46]. In clinical studies, myelin has been reported to be involved in MDD [47, 48]. Demyelination has also been reported to occur in various depression-like animal models in preclinical studies [49, 50]. Liu et al. [13] suggested that clemastine, a myelination-promoting drug, alleviates depression-like behaviors in socially isolated mice. Physical exercise has been reported to improve the cognitive function of rats with chronic cerebral hypoperfusion and MS, possibly by enhancing oligodendrogenesis and remyelination in the hippocampus [51, 52]. Moreover, in the current study, running exercise significantly reversed the decrease in the percentage of MBP^+^ myelinated fibers and the thickness of the myelin sheath in the CA1 hippocampal subfield in the UCS-exposed depression model mice (Fig. 6a–d). We speculate that the preservation of myelination is a potential therapeutic strategy for depression. In this study, we showed for the first time that UCS suppressed OPC differentiation and myelination and that this effect was attenuated by running exercise. In contrast to running exercise, fluoxetine failed to reverse the changes in oligodendrocyte maturation and differentiation as well as the decrease in the percentage of MBP^+^-myelinated fibers and the thickness of the myelin sheath in the hippocampi of UCS-exposed depression model mice. Rajkowska et al. [17] found that the chronic treatment of rhesus monkeys with fluoxetine had no significant effect on oligodendrocyte morphometry. In other previous studies, running exercise was found to protect myelinated fibers in the white matter of a UCS-induced rat model of depression, while fluoxetine had no effect on white matter myelinated fibers [6, 53]. We observed notable differences in myelination between the fluoxetine and exercise treatment groups in our previous study and in the current study. Why did fluoxetine fail to promote these changes in oligodendrocyte biology and myelination? A previous study found that the efficacy of fluoxetine in a depression model depended on both the activity and density of cell surface-expressed serotonin transporters and 5-HT autoreceptors [54]. 5-HT receptors are expressed primarily on neurons and not on oligodendrocytes [55]. Many studies have suggested that promoting the differentiation and myelination of oligodendrocytes can reverse depression-like behavior [13, 56]. Therefore, we speculate that the promotion of oligodendrocyte differentiation and subsequent myelination in the hippocampus is a mechanism by which running exercise, but not fluoxetine, reverses depression-like behavior in UCS-exposed mice. However, further experiments are needed to confirm this mechanism.

Notably, the changing trend in OPC numbers in the current study contrasts with those reported in the literature. In our study, the number of CC1^+^ mature oligodendrocytes in the hippocampus was decreased in UCS mice; however, the number of PDGFα^+^ OPCs was increased. Moreover, running exercise significantly reversed these UCS-induced changes (Figs. 3, 4, 5). In previous studies on animal models of major depression, stressful experiences were found to decrease the density of OPCs and the proliferation of oligodendrocytes in the frontal cortex and amygdala [57, 58]. However, opposite findings regarding the number of OPCs were reported in another recent study. Liu et al. [13] revealed a significant increase in the number of OPCs (NG2^+^ cells) in the prefrontal cortices of depressed mice compared with their littermate controls. We found an abnormal increase in the number of hippocampal OPCs in depression model mice by using a more objective and accurate cell counting method in the current study. Many studies found that exercise promoted OPC proliferation in healthy mice and in subjects with other diseases, as the number of OPCs in healthy mice and in rats with ischemia was not abnormally increased [59, 60]. However, we herein found that the OPC population was increased and that the mature oligodendrocyte population was decreased in mice treated with UCS. Two factors may underlie the increase in the OPC population: (1) UCS may promote OPC proliferation rather than differentiation, or (2) OPCs may stay in the G1 phase longer or exit the cell cycle but not differentiate at the usual rate. In addition, we herein found that running exercise may reduce the number of OPCs and increase that of mature oligodendrocytes by decreasing OPC proliferation and promoting the differentiation of OPCs into mature oligodendrocytes. We speculated that the mechanism by which running exercise promotes or decreases OPC proliferation depends on the demands of the various pathogeneses.

In the current study, the proliferation of OPCs was increased in both the CA1 and DG subfields of the hippocampus; however, the differentiation and maturation of oligodendrocytes and myelination were decreased in only the CA1 hippocampal subfield in the UCS-exposed depression model mice. In addition, running exercise rather than fluoxetine promoted the differentiation of OPCs into mature oligodendrocytes and subsequent myelination in only the CA1 subfield of the hippocampus. To our knowledge, no data are available that directly illustrate the different stages of oligodendrocyte changes from OPCs to myelination in the three subfields of the hippocampus in UCS-exposed depression model mice treated with running exercise or fluoxetine. The hippocampus is a uniform structure composed of the following subregions with different morphologies: the presubiculum, adjacent subiculum, fimbria, DG subfield, and CA subfield [61]. Moreover, the DG subfield contains the hilus, granular layer, and molecular layer [62]. In the current study, the number of OPCs was increased in only the hilus of the DG subfield and not in the molecular layer, and numerous OPCs accumulated in the hilus; specifically, more than twice the number of OPCs accumulated in the hilus of the DG subfield than in the other subfields of the hippocampus (Fig. 5c). Yuliana et al. [63] found that 84% of proliferating cells in the hilus corresponded to neural precursor cells, of which OPCs were the most abundant, accounting for 54%, whereas OPCs accounted for only 11% in the subgranular zone. Stimulation of human mesenchymal stem cell (MSC) factors was shown to equally promote the oligodendrogenesis of rat stem cells, axon encapsulation, and tissue integration in the hilus of the hippocampus [64]. Based on these results, the proliferation of OPCs in the hilus of the DG subfield may play an important role in UCS-exposed depression in mice. However, the proliferation of OPCs was significantly increased in the DG subfield of the UCS-exposed depression-like model mice, whereas the numbers of myelinated and mature oligodendrocytes were not changed in the DG subfield. Unlike in the DG subfield, the differentiation and maturation of oligodendrocytes were decreased, and myelination was reduced in the CA1 subfield of mice with depression-like behaviors induced by UCS; however, running exercise ameliorated these changes (Fig. 4 and Fig. 6). In a clinical study, overall changes were most marked in the left CA1, and the CA1 volume was a predictor of MDD [65]. In another study, abnormal microstructures of CA2-3 and CA1 were related to generalized anxiety disorder/MDD comorbidity [66]. The hippocampal CA1 region was previously shown to be closely associated with the pathogenesis of depression in preclinical studies [67–69]. Thus, we speculate that the CA1 hippocampal subfield might be more sensitive to the pathogenesis of depression. In the current study, running exercise, not fluoxetine, promoted OPC differentiation and myelination in the CA1 subfield, thereby potentially overcoming the deficits in this region of the hippocampus and subsequent behavioral deficits. Based on the results described above, we speculated that the exposure of mice to UCS caused OPCs stored in the DG subfield to enter their proliferation cycle. The OPCs then migrated to damaged hippocampal subfields, such as the CA1 subfield, accounting for the increased numbers of OPCs in the CA1 and DG subfields of UCS mice. Due to chronic stress, the proliferating OPCs in the CA1 subfield could not differentiate into mature oligodendrocytes and subsequently undergo myelination. However, the mechanism and implication of hippocampal region-specific changes reported in the current study need to be further investigated.

In summary, we propose that running exercise treatment reverses depression-like behavior in UCS-exposed mice faster and more efficiently than fluoxetine treatment, possibly by enhancing oligodendrocyte differentiation and myelination in the CA1 subfield of the hippocampus and contributing to the amelioration of behavioral changes in the mice. Therefore, our findings provide a structural basis for the role of oligodendrocyte function and myelination in regulating depression-like behaviors and may contribute to the discovery of new strategies for the treatment of depression.

### Limitations

The ethical and practical limitations of directly studying the human brain make animal models a necessary tool for identifying depression mechanisms and exploring novel therapeutics for depression. However, no animal model can perfectly recapitulate depression. Moreover, in the current study, biological changes were measured at only a single time point at the end of the study and therefore represent a snapshot. We do not know what changes occurred in these parameters at early time points during the 65-day experiment. To ensure that the effects observed in treated animals were due to the intervention itself and not to other potential confounders, studies should be performed with three control groups to examine the effects of fluoxetine and exercise on both control and stressed animals. The lack of these groups was a limitation of the experimental design utilized in the current study. Two additional important groups will be considered for inclusion in our future experimental design. Another limitation of this study was the use of only male mice, as twice as many women experience major depression than men [70]. Many studies have found that antidepressant therapies in female models of depression are affected by many factors, such as the estrus cycle and estrogen [71–73]. Therefore, the effects of exercise and fluoxetine on the oligodendrocyte differentiation and myelination of the hippocampus in a female mouse model of depression need to be further investigated.

## Supplementary information

SUPPLEMENTAL MATERIAL
