# Supplementary material for: Exercise rather than fluoxetine promotes oligodendrocyte differentiation and myelination in the hippocampus in a male mouse model of depression
Source: Transl Psychiatry. 2021 Dec 8;11:622. doi: 10.1038/s41398-021-01747-3 (PMC8654899; doi:10.1038/s41398-021-01747-3)
Supplement: Supplementary file 1 — SUPPLEMENTAL MATERIAL [file 41398_2021_1747_MOESM1_ESM.docx]

**Title:** Exercise Rather Than Fluoxetine Promotes Oligodendrocyte Differentiation and Myelination in the Hippocampus in a Male Mouse Model of Depression

**Supplemental Information**

**Materials and Methods**

**Animal housing and UCS**

Sixty male C57BL/6J mice were used in the present study. The mice (10–12 weeks old at the beginning of the UCS exposure) were housed in groups of 5 per cage on a 12-h light/dark cycle (lights on at 07:00 a.m.) at a constant temperature (22 ± 1°C) and humidity with free access to food and water. The animals were allowed 1 week to habituate to the housing conditions before any experiments were initiated. All animals were age- and weight-matched (the mice weighed 10–15 g) at the beginning of the experiments. All mouse housing, treatment and maintenance procedures were performed in accordance with the National Institutes of Health Guide for the Care and Use of Laboratory Animals and were consistent with the Chongqing Medical University Care and Use of Laboratory Animals guidelines.

**UCS paradigm**

After a week of adjustable feeding, the mice were randomly divided into a CTRL group (n =14) and a UCS standard group (n = 46). The mice in the CTRL group did not receive any treatments but were handled daily. The mice in the UCS group were housed at a density of one animal per cage and exposed to UCS stimulation for four weeks. Briefly, the animals were exposed to three stressors each day–one in the morning, one in the afternoon, and one overnight–for 12 consecutive days in dedicated procedure rooms. This procedure was adapted from those in studies by Willner et al. ^1^, Yohn et al. ^2^, and Logan et al. ^3^ (Tables 1-2). After 4 weeks of UCS exposure, 4 mice in the UCS standard group were removed because they displayed a side preference in the SPT, and the mice in the UCS standard group (n=42) were randomly divided into a UCS group (n = 14), a UCS + running group (n = 14) and a UCS + FLX group (n=14). The effects of stress on the hedonic states of the mice were assessed weekly using SPT and BW measurements (Fig. 1a). This study was approved by the Animal Care and Research Committee of Chongqing Medical University, P. R. China.

**Exercise protocol**

The mice in the running group were placed on a treadmill to run regularly for four months Based on a previous study, the exercise regimen used herein, involving a velocity increase of 10 m/min (20 min per day, 5 days per week), led to approximately 60% of the VO_2max_ being achieved; thus, the exercise was equivalent to moderate intensity and was not stressful for the C57BL/6J male mice ^4,5^. During the first two weeks, which was the adaptation period, a running speed of 5 m/min was gradually increased to 10 m/min. For the rest of the experiment, the running speed was maintained at 10 m/min (20 min per day, 5 days per week).

**Fluoxetine and BrdU injections**

The mice in the UCS + FLX group were intraperitoneally injected with fluoxetine (Sigma-Aldrich, USA) at a dosage of 10 mg/kg/d (time of administration: 10:30–11:00 a.m.) once daily from day 28 to day 56 during the UCS period (a total of 28 days) ^6^.

The mice were intraperitoneally injected with BrdU (50 μg/g BW, dissolved in 0.9% saline at a concentration of 10 mg/mL; Sigma-Aldrich, USA) once daily from day 28 to day 42 (a total of 14 days) to study the differentiation of oligodendrocytes in the three subfields of the hippocampus ^4^.

**BW measurement**

BW measurements were obtained on the first day and before sucrose consumption every week.

**SPT**

The SPT was performed using a method previously described by Tang et al. ^7^. All animals, including littermate controls, were individually housed during the SPT. To facilitate the adaptation of the control animals to the housing conditions for the SPT, the mice in the control group were individually housed 12 h before the SPT during the adaptation period. Sucrose and water consumption were determined by measuring the change in the volume of each fluid after consumption. The animals were first trained with both bottles while housed in groups. To prevent side preferences, the bottle location of the sucrose solution was changed from the left side to the right side every 12 h during this test. The volumes of water and sucrose consumed were measured by weighing the bottles. A loss of preference for sucrose suggested anhedonia, a core symptom of patients with depression.

**FST**

The FST was conducted as previously described ^4,8^. Before the FST, the mice were moved to the animal room adjacent to the test room in which their behaviors were assessed and housed there for 1 week under the same conditions as those before transport. The mice were assayed during the light cycle (11:00–16:00) after adapting to the test room for at least 2 h. The mice were placed in transparent Plexiglas cylinders (20-cm diameter × 30-cm high) containing water (23°C ± 1°C) to a depth of 15 cm. The mice were kept in the cylinders for a period of 6 min, and the time spent immobile during the last 4 min of the 6-min period was scored. A recent study suggested that the FST can be used more than once and that mice show a consistent individual pattern of responding in the test^9^. Therefore, in our study, the FST was performed after UCS and then again at the end of the study.

**TST**

The TST was conducted as previously described ^4,10^. Before the TST, the mice were moved to the animal room adjacent to the test room in which their behaviors were assessed and housed there for 1 week under the same conditions as those before transport. The mice were assayed during the light cycle (11:00–16:00) after adapting to the test room for at least 2 h. The mice were suspended by their tails using an elastic band (5 cm in diameter) attached to the tail with adhesive tape (approximately 1 cm from the tip of the tail), and the elastic band was hooked onto a horizontal rod. The distance between the tip of the nose of the mouse and the floor was approximately 20 cm. The mice were suspended by their tails in transparent plastic cages (35 cm × 19 cm × 13 cm). The mice were suspended for a period of 6 min, and the time spent immobile during the last 4 min of the 6-min period was scored. the TST was performed only once at the end of the study.

**Perfusion and tissue preparation**

In the present study, fourteen mice were randomly selected from each group and used for immunohistochemical (5 mice in each group), immunofluorescence (5 mice in each group) and electron microscopy (EM; 4 mice in each group) analyses. The animals were sacrificed 12 h after the behavioral test. The animals were anesthetized by an i.p. injection of 1% pentobarbital sodium (0.4 mL/100 g) and then perfusion-fixed with 2% paraformaldehyde and 2.5% glutaraldehyde in 0.1 M phosphate-buffered saline (PBS) at pH 7.4. After each animal was perfusion-fixed, the cerebrum, meninges, cerebellum and brain stem were removed. The right or left hemisphere was chosen at random prior to sectioning. The hemispheres were cut into 50-µm-thick serial sections, and every fifth section was sampled for analysis in a systematic random manner. On average, 15–18 hippocampal sections were sampled from each hemisphere. Prior to all staining procedures, the vibratome and cryostat sections were rinsed several times with 0.01 M PBS (pH 7.4) to remove all sucrose and glycerol left over from the cryopreservation solution. The protocol was previously described by Tang et al. ^7^.

**Immunohistochemistry and stereological cell counting**

Two separate sets of serial hippocampal sections from each group of mice were chosen and subjected to immunostaining with goat anti-PDGFα (1:500; AF1062, R&D Systems) and mouse anti-CC1 (1:500; OP80, EMD Millipore) antibodies for stereological analyses of the total numbers of OPCs and mature oligodendrocytes in the hippocampus. The sections were stained using an Anti-Mouse HRP-DAB Cell & Tissue Staining Kit (brown; SP-9002, Beijing Noble Ryder Technology) and an Anti-Goat HRP-DAB Cell & Tissue Staining Kit (brown; PV-9003, Beijing Noble Ryder Technology). Prior to all staining procedures, the vibratome and cryostat sections were rinsed several times with 0.01 M PBS (pH 7.4) to remove all sucrose and glycerol remaining from the cryopreservation solution. The immunohistochemistry protocol was initiated by rinsing with PBS containing 0.3% Triton X-100 (cat #X100, Sigma-Aldrich) and 0.1% Tween (PBS + T). Unless otherwise stated, all washes and incubations were performed in 6-well Netwell plates at room temperature (RT, 21–23°C) with gentle agitation. Free-floating sections were blocked with 1% fetal bovine serum (FBS), 10% SP 9002-A, and PBS+T and then incubated with primary antibodies for two days at 4°C. The next morning, the sections were rewarmed at 37°C for 1 h. The sections were washed with PBS again before being incubated with SP 9002-B/PV 9003-A in water for 3 h at 37°C and then with SP 9002-C/PV 9002-B for 2 h at 37°C. Then, the sections were transferred to a diaminobenzidine (DAB) solution (DAB, ZLL-9032, ZSGB; China) for approximately 1 min. Finally, the sections were dehydrated by sequential immersion in a graded ethanol series (70%, 80%, 90%, 100%, and 100%; 10 min each) and xylene (3 × 10 min). An optical fractionator was used to estimate the total numbers of PDGFα^+^ and CC1^+^ cells in the hippocampus.

The hippocampal subfields were delineated based on cytoarchitectonic criteria ^7,11^. The numbers of PDGFα^+^ and CC1^+^ cells in the three hippocampal subfields were estimated using the optical fractionator method. Briefly, the stereological system with an integrated hardware/software setup consisted of a personal computer running the Visiopharm Integrator System (VIS, Denmark) and a microscope (Olympus, BX51, Japan). The computer controlled the 3-axis motorized stage on the microscope with a resolution of 0.1 μm, enabling fully automated sampling and z-depth monitoring. Images from the microscope were captured using a digital camera (Olympus, DP71, Japan) and superimposed onto the screen with computer-generated graphical representations of the sampling area borders, counting grids, and counting particles.

In the present study, 50-μm-thick immunohistochemically stained sections were used as guides for the accurate delineation of the three areas in the sections. Sampling at every stage was performed randomly and systematically. Thus, each section had an equal probability of being sampled, and the interval between each section and each counting site was constant. The variable *ssf* is the section sampling fraction, *asf* is the area sampling fraction, and *hsf* is the thickness sampling fraction. From these parameters and the number of cells actually counted in the specimens (ΣQ^−^), the total number of cells (N) was estimated using the following equation.

$$N=\sum Q^{-}\times\frac{1}{ssf}\times\frac{1}{asf}\times\frac{1}{hsf}$$

For example, the optical fractionator method was used to estimate the total number of CC1^+^ cells (Fig. S1). Every fifth section was sampled for analysis using a random starting point in the series. Therefore, *ssf* was 1/5. Subsequently, the ratio between the area of the unbiased counting frame and the rectangular area that was obtained by multiplying the step length in the x-axis and the step length in the y-axis represented the area sampling fraction (*asf*), which was 6% in the CA1 and DG subfields and 8% in the CA3 subfield. The mean thickness of each section was approximately 30 μm (t, section thickness). The top of the counting frame in the z-dimension was set to 3 μm from the section surface (guard zone). Thereafter, these positive cells were counted while focusing at 15 μm through the section (h, disector height). The third fraction, *hsf*, was therefore calculated as h/t = 15/22. The solid line of the frame and its extension represent the exclusion lines, and the dotted line of the frame represents the inclusion line. When the inclusion (dotted) line was green, the CC1^+^ cells (indicated by the green arrow) that were completely inside the counting frame or partially inside the counting frame but only touching the inclusion (dotted) line were counted (Fig. S1). When the optical fractionator method was used to estimate the total number of PDGFα^+^ cells, *ssf* was 1/5; a*sf* was 6% in the CA1, CA3 and DG subfields; and *hsf* was approximately 15/19.

**Immunofluorescence analyses**

Mice were anesthetized and then perfused; their tissues were cryopreserved, embedded, and sectioned as described above. Immunohistochemistry was performed with primary antibodies against PDGFα (1:500; AF1062, R&D Systems), CC1 (1:500; OP80, EMD Millipore), BrdU (1:500; ab6326, Abcam), MBP (1:1000; ab62631, Abcam), and Olig2 (1:500; ab109186, Abcam). The secondary antibodies included goat anti-rat IgG (DyLight 408, 1:100; Abbkine, USA), goat anti-mouse IgG (DyLight 488, 1:100; Abbkine, USA), goat anti-rabbit IgG (DyLight 549, 1:100; Abbkine, USA) and IFKine red donkey anti-goat IgG (H+L) (1:100; Abbkine, USA). The immunofluorescence protocol was initiated by rinsing with PBS+T, after which free-floating sections were blocked with 1% FBS, 10% SP 9002-A, and PBS+T and then incubated with primary antibodies for two days at 4°C. The next morning, the sections were rewarmed at 37°C for 1 h. After rewashing with PBS, the sections were incubated with the appropriate secondary antibodies for 2 h at 37°C, washed with PBS for 30 min (10 min × 3), and incubated with DAPI (AR1177, Boster, China). The stained sections were visualized using a laser scanning confocal microscope (Nikon Eclipse Ti microscope, NIS-Elements AR Imaging). For the quantification of PDGFα^+^/Olig2^+^ and CC1^+^/Olig2^+^/BrdU^+^ cells, images of the whole hippocampus were captured using the Grab Large Image tool, and a rectangular grid was superimposed onto the hippocampus. Then, 12–15 fields (100 × 100 μm^2^) were systematically and randomly sampled from all fields in the CA1, CA3 and DG regions of mice. For example, the counted field shown in Figure 1c was magnified from the blue grid (100 × 100 μm^2^) of the CC1^+^/Olig2^+^/BrdU^+^ cells shown in Figure 1b. To quantify the MBP^+^ area, images of 4–6 fields (320 × 320 μm^2^) of the CA1, CA3 and DG regions of each mouse were captured using a laser scanning confocal microscope and a 20 × objective. Five mice were included per treatment condition. The CC1^+^/Olig2^+^/BrdU^+^ and PDGFα^+^/Olig2^+^ cells were quantified using the count quantification tool of the NIS-Elements AR analysis system. The MBP^+^ areas were quantified using the defined area method of the NIS-Elements AR analysis system.

**EM and G-ratio analysis**

The cerebrum was excised and divided into 2 hemispheres along the mid-sagittal plane. Each hemisphere was perfused with 0.1 M PB followed by 2% glutaraldehyde/4% paraformaldehyde (PFA) in sodium cacodylate buffer. A plastic sheet with equidistant points was placed randomly on the surface of the sampled slabs. A block of approximately 1×1×1 mm^3^ from the CA1 hippocampal region was dissected, postfixed overnight at 4°C, and then contrasted with 1% osmic acid (vol/vol) in PBS. Five tissue blocks per mouse were sampled at random. For EM, the mounted section was treated with 4% glutaraldehyde for at least 2 hours at 4°C and rinsed three times with 0.1 M PBS. The tissues were embedded in 5-mm spheres with Epon. After hardening, the spheres were rotated randomly and then re-embedded. This procedure, termed the isector method ^12^, ensures the selection of isotropic, uniform, and random sections such that each tissue sample has a uniformly random orientation before sectioning. The ultrathin sections were then viewed under a transmission electron microscope (Hitachi-7500, Hitachi, Ltd., Japan). Twenty fields of view in each section were randomly chosen and photographed with a transmission electron microscope at a magnification of 10000× (Fig. 2). One hundred to 125 EM images were obtained from each group. ImageJ analysis software was used to measure the axon diameter and outer diameter of each myelinated nerve fiber in the CA1 hippocampal region, and the G-ratio value was calculated using the following formula:

G-ratio = axon diameter of myelinated nerve fibers/outer myelin diameter of myelinated nerve fibers

**Figure**

Figure S1


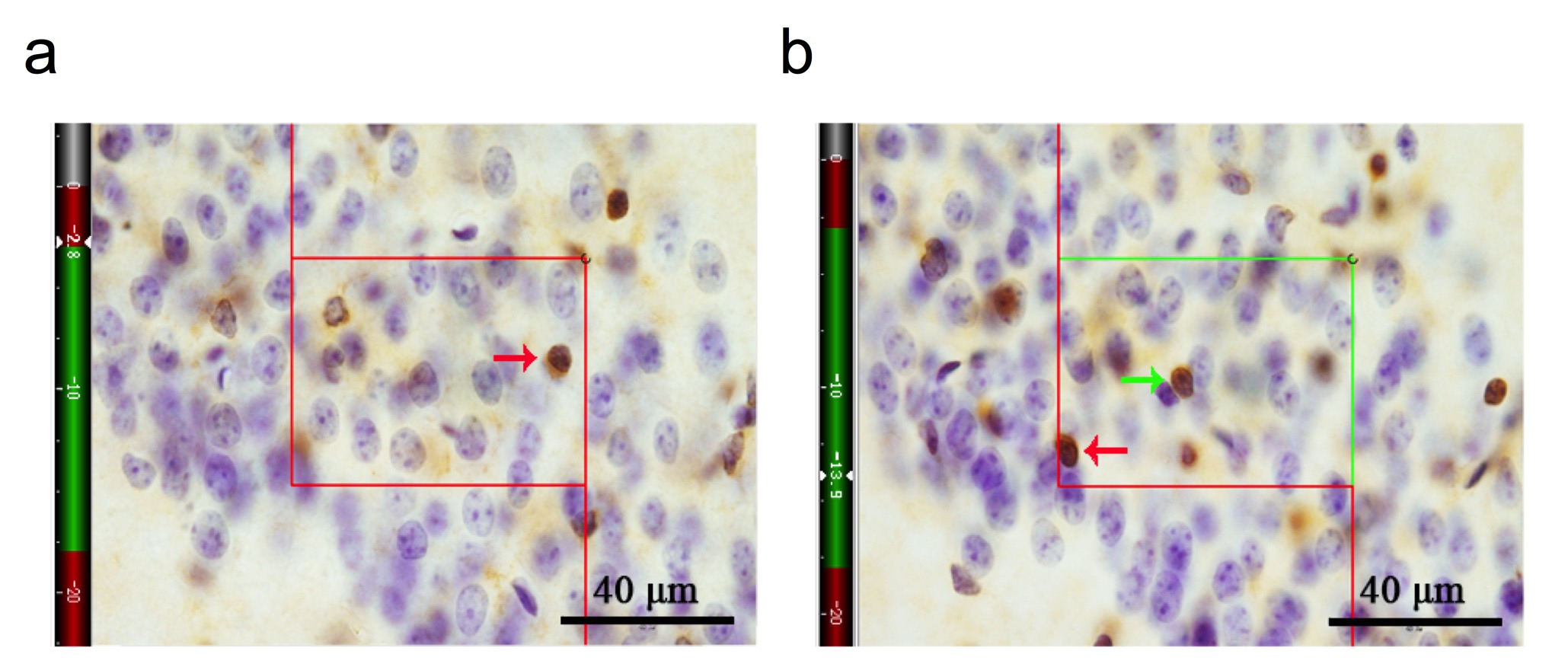


**Figure S1.** Illustration of the stereological method used to count the CC1^+^ cells. **(a)** CC1^+^ cells with clear nuclei in the guard zone were not counted, as indicated by the red arrow. **(b)** CC1^+^ cells with clear nuclei outside the counting frame or touching the red lines were not counted, as indicated by the red arrow. CC1^+^ cells with clear nuclei that were completely inside the counting frame or partially inside the counting frame touching the green lines but not touching the red lines were counted, as indicated by the green arrow. Scale bar = 40 μm. The top of the counting frame in the z-dimension was set to 3 μm from the section surface (guard zone). Starting at 3 μm from the top surface of the sections, the CC1^+^ cells were counted while focusing at 15 μm through the sections, i.e., within the range of 3–18 μm from the section surface.

Table S1. Stereological estimation of PDGFα^+^ cell numbers

|  |  | CTRL (n = 5) | | UCS (n = 5) | | UCS + RN (n = 5) | | UCS + FLX (n = 5) | |
| --- | --- | --- | --- | --- | --- | --- | --- | --- | --- |
|  |  | THK  (μm) | N (×10^4^) | THK  (μm) | N (×10^4^) | THK (μm) | N (×10^4^) | THK  (μm) | N (×10^4^) |
| CA1 | 1 | 17.56 | 1.28 | 17.24 | 1.62 | 17.81 | 1.14 | 17.32 | 1.07 |
|  | 2 | 19.68 | 1.59 | 17.43 | 2.12 | 17.84 | 1.45 | 17.49 | 1.71 |
|  | 3 | 17.21 | 1.39 | 17.49 | 1.59 | 17.22 | 1.42 | 17.47 | 1.77 |
|  | 4 | 18.06 | 1.58 | 17.30 | 1.83 | 17.32 | 1.38 | 17.48 | 1.19 |
|  | 5 | 17.21 | 1.58 | 17.50 | 1.76 | 17.59 | 1.30 | 18.99 | 1.52 |
|  | Mean | 17.94 | 1.48 | 17.39 | 1.79 | 17.56 | 1.34 | 17.75 | 1.45 |
|  | SD | 1.03 | 0.14 | 0.12 | 0.21 | 0.28 | 0.12 | 0.70 | 0.31 |
|  | OCV | 0.10 | | 0.12 | | 0.09 | | 0.22 | |
|  | OCE | 0.05 | | 0.05 | | 0.05 | | 0.05 | |
|  | OCE^2^/  OCV^2^ | 0.22 | | 0.14 | | 0.25 | | 0.06 | |
| CA3 | 1 | 17.88 | 0.66 | 17.43 | 0.91 | 17.52 | 0.82 | 17.47 | 0.93 |
|  | 2 | 19.57 | 1.06 | 17.37 | 1.05 | 17.78 | 0.89 | 17.97 | 0.88 |
|  | 3 | 17.25 | 0.79 | 17.90 | 1.23 | 17.41 | 1.07 | 17.57 | 1.04 |
|  | 4 | 17.62 | 0.84 | 17.43 | 1.04 | 17.71 | 0.99 | 17.52 | 0.97 |
|  | 5 | 17.83 | 0.89 | 17.59 | 1.14 | 18.08 | 0.86 | 18.64 | 0.81 |
|  | Mean | 18.03 | 0.87 | 17.54 | 1.07 | 17.70 | 0.93 | 17.83 | 0.93 |
|  | SD | 0.90 | 0.16 | 0.22 | 0.12 | 0.26 | 0.10 | 0.49 | 0.09 |
|  | OCV | 0.19 | | 0.11 | | 0.11 | | 0.10 | |
|  | OCE | 0.07 | | 0.06 | | 0.06 | | 0.07 | |
|  | OCE^2^/  OCV^2^ | 0.11 | | 0.23 | | 0.25 | | 0.33 | |
| DG | 1 | 17.72 | 0.93 | 17.52 | 1.13 | 17.27 | 0.98 | 17.47 | 0.88 |
|  | 2 | 19.50 | 1.22 | 17.26 | 1.50 | 17.74 | 0.87 | 17.85 | 1.03 |
|  | 3 | 17.54 | 1.18 | 17.53 | 1.43 | 17.83 | 1.18 | 18.21 | 1.01 |
|  | 4 | 17.28 | 1.29 | 17.26 | 1.41 | 17.91 | 1.00 | 17.36 | 0.75 |
|  | 5 | 17.73 | 1.23 | 17.50 | 1.19 | 18.66 | 1.08 | 17.49 | 1.14 |
|  | Mean | 17.95 | 1.13 | 17.41 | 1.33 | 17.88 | 1.02 | 17.68 | 0.96 |
|  | SD | 0.88 | 0.15 | 0.14 | 0.16 | 0.50 | 0.12 | 0.35 | 0.15 |
|  | OCV | 0.13 | | 0.12 | | 0.11 | | 0.16 | |
|  | OCE | 0.05 | | 0.04 | | 0.05 | | 0.05 | |
|  | OCE^2^/  OCV^2^ | 0.12 | | 0.11 | | 0.16 | | 0.09 | |

*The mean value of each variable (mean), standard deviation (SD), observed coefficient of variation (OCV) and observed coefficient of error (OCE) are provided. THK: thickness; N: total number; n: sample size*

Table S2. Stereological estimation of CC1^+^ cell numbers

|  |  | CTRL (n = 5) | | UCS (n = 5) | | UCS + RN (n = 5) | | UCS + FLX (n = 5) | |
| --- | --- | --- | --- | --- | --- | --- | --- | --- | --- |
|  |  | THK  (μm) | N (×10^4^) | THK  (μm) | N (×10^4^) | THK (μm) | N (×10^4^) | THK  (μm) | N (×10^4^) |
| CA1 | 1 | 19.70 | 2.68 | 17.93 | 1.78 | 24.78 | 3.14 | 23.56 | 2.70 |
|  | 2 | 19.11 | 2.75 | 24.53 | 1.84 | 18.31 | 2.18 | 23.02 | 3.10 |
|  | 3 | 18.32 | 3.20 | 19.42 | 2.34 | 23.02 | 3.09 | 21.25 | 2.17 |
|  | 4 | 21.49 | 2.81 | 20.62 | 2.58 | 20.02 | 3.43 | 19.39 | 2.95 |
|  | 5 | 21.00 | 2.99 | 19.35 | 2.56 | 20.75 | 2.46 | 20.10 | 2.61 |
|  | Mean | 19.92 | 2.89 | 20.37 | 2.22 | 21.38 | 2.86 | 21.46 | 2.70 |
|  | SD | 1.17 | 0.21 | 2.25 | 0.38 | 2.28 | 0.52 | 1.61 | 0.36 |
|  | OCV | 0.07 | | 0.17 | | 0.18 | | 0.13 | |
|  | OCE | 0.06 | | 0.07 | | 0.07 | | 0.07 | |
|  | OCE^2^/  OCV^2^ | 0.42 | | 0.15 | | 0.19 | | 0.12 | |
| CA3 | 1 | 20.05 | 2.93 | 18.44 | 3.07 | 24.00 | 4.24 | 23.28 | 3.31 |
|  | 2 | 20.33 | 3.50 | 24.56 | 2.76 | 19.63 | 3.67 | 21.16 | 4.03 |
|  | 3 | 19.31 | 3.28 | 19.47 | 3.91 | 21.63 | 3.51 | 22.33 | 3.09 |
|  | 4 | 31.28 | 3.42 | 20.57 | 3.89 | 19.97 | 3.84 | 18.86 | 3.34 |
|  | 5 | 21.38 | 3.27 | 19.38 | 3.10 | 21.63 | 3.50 | 22.28 | 3.62 |
|  | Mean | 22.47 | 3.26 | 20.48 | 3.35 | 21.37 | 3.75 | 21.58 | 3.48 |
|  | SD | 4.45 | 0.22 | 2.15 | 0.52 | 1.55 | 0.31 | 1.52 | 0.46 |
|  | OCV | 0.07 | | 0.16 | | 0.08 | | 0.10 | |
|  | OCE | 0.06 | | 0.06 | | 0.06 | | 0.06 | |
|  | OCE^2^/  OCV^2^ | 0.45 | | 0.12 | | 0.24 | | 0.32 | |
| DG | 1 | 19.54 | 1.37 | 17.82 | 1.53 | 24.49 | 1.59 | 22.97 | 1.90 |
|  | 2 | 19.30 | 1.81 | 23.99 | 1.73 | 18.68 | 1.73 | 19.47 | 2.00 |
|  | 3 | 18.22 | 1.85 | 18.31 | 1.73 | 20.94 | 2.18 | 21.60 | 1.81 |
|  | 4 | 30.47 | 2.10 | 19.79 | 2.09 | 19.49 | 2.55 | 18.57 | 2.49 |
|  | 5 | 19.55 | 1.76 | 18.43 | 1.55 | 19.62 | 1.59 | 22.37 | 1.80 |
|  | Mean | 21.42 | 1.78 | 19.67 | 1.73 | 20.64 | 1.93 | 21.00 | 2.00 |
|  | SD | 4.55 | 0.26 | 2.26 | 0.22 | 2.06 | 0.42 | 1.69 | 0.29 |
|  | OCV | 0.15 | | 0.13 | | 0.22 | | 0.14 | |
|  | OCE | 0.05 | | 0.06 | | 0.05 | | 0.05 | |
|  | OCE^2^/  OCV^2^ | 0.11 | | 0.15 | | 0.10 | | 0.05 | |

*The mean value of each variable (mean), standard deviation (SD), observed coefficient of variation (OCV) and observed coefficient of error (OCE) are provided. THK: thickness; N: total number; n = sample size*

**References**

1 Willner P, Towell A, Sampson D, Sophokleous S, Muscat R. Reduction of sucrose preference by chronic unpredictable mild stress, and its restoration by a tricyclic antidepressant. *Psychopharmacol* 1987; **93**: 358–364.

2 Yohn NL, Blendy JA. Adolescent chronic unpredictable stress exposure is a sensitive window for long-term changes in adult behavior in mice. *Neuropsychopharmacology* 2017; **42**: 1670–1678.

3 Logan RW, Edgar N, Gillman AG, Hoffman D, Zhu X, McClung CA. Chronic stress induces brain region-specific alterations of molecular rhythms that correlate with depression-like behavior in mice. *Biol Psychiatry* 2015; **78**: 249–258.

4 Wang J, Tang J, Liang X, Luo Y, Zhu P, Li Y *et al.* Hippocampal PGC-1α-mediated positive effects on parvalbumin interneurons are required for the antidepressant effects of running exercise. *Transl Psychiatry* 2021; **11**: 222.

5 Yook JS, Rakwal R, Shibato J, Takahashi K, Koizumi H, Shima T *et al.* Leptin in hippocampus mediates benefits of mild exercise by an antioxidant on neurogenesis and memory. *Proc Natl Acad Sci U S A* 2019; **116**: 10988–10993.

6 Sales AJ, Crestani CC, Guimaraes FS, Joca SRL. Antidepressant-like effect induced by Cannabidiol is dependent on brain serotonin levels. *Prog Neuropsychopharmacol Biol Psychiatry* 2018; **86**: 255–261.

7 Tang J, Liang X, Zhang Y, Chen L, Wang F, Tan C *et al.* The effects of running exercise on oligodendrocytes in the hippocampus of rats with depression induced by chronic unpredictable stress. *Brain Res Bull* 2019; **149**: 1–10.

8 Porsolt RD, Le Pichon M, Jalfre M. Depression: a new animal model sensitive to antidepressant treatments. *Nature* 1977; **266**: 730–732.

9 Kazavchinsky L, Dafna A, Einat H. Individual variability in female and male mice in a test-retest protocol of the forced swim test. *J Pharmacol Toxicol Methods* 2019; **95**: 12–15.

10 Steru L, Chermat R, Thierry B, Simon P. The tail suspension test: a new method for screening antidepressants in mice. *Psychopharmacology (Berl)* 1985; **85**: 367–370.

11 Buhl EH, Dann JF. Cytoarchitecture, neuronal composition, and entorhinal afferents of the flying fox hippocampus. *Hippocampus* 1991; **1**: 131–152.

12 Nyengaard JR, Gundersen HJG. The isector: a simple and direct method for generating isotropic, uniform random sections from small specimens. *J Microsc* 1992; **165**: 427–431.
